# Endoscopic Totally Extraperitoneal Repair of Parastomal Hernia: A Case Report

**DOI:** 10.3389/fsurg.2021.659102

**Published:** 2021-05-20

**Authors:** Huiyong Jiang, Dil Momin Thapa, Chun Ma, Xiangjun Cai, Mofei Wang

**Affiliations:** ^1^The Second Department of General Surgery, Northeast International Hospital, Shenyang, China; ^2^Clinical Medical School of Inner Mongolia University for the Nationalities, Tongliao, China; ^3^The Second Department of General Surgery, The Affiliated Hospital of Inner Mongolia University for the Nationalities, Tongliao, China

**Keywords:** endoscopy, parastomal hernia, surgical repair, totally extraperitoneal repair, rectal cancer

## Abstract

A parastomal hernia is a type of incisional hernia that occurs in abdominal integuments in the proximity of a stoma. It is a frequent late complication following colostomy. Surgical repair is currently the only treatment option for parastomal hernia. Here we present the case of a 74-year-old patient with parastomal hernia and a history of open surgery treated with a totally extraperitoneal (TEP) endoscopic approach. There was no recurrence of the hernia at the 3-month follow-up. We discuss the feasibility and possible operative approaches for endoscopic repair of parastomal hernia with the TEP technique.

## Introduction

Parastomal hernia, a frequent late complication following colostomy, involves protrusion of the abdominal viscera through a defect in the abdominal wall around the stoma; it has a high incidence rate (30–50%) (1–4) and is difficult to treat, with a high recurrence rate after repair (15.7%) (5, 6). Parastomal hernia of the sigmoid colon through the abdominal wall and perineum after radical resection of rectal carcinoma is common. The incidence of parastomal hernias is increasing with the prolongation of patient survival after this surgery; in severe cases, incarceration or intestinal obstruction may be life-threatening.

Surgical repair is the treatment of choice for parastomal hernia (7). Surgical approaches commonly employed in the past such as pure fascia repair and stoma relocation have been abandoned as they yielded poor results. Repair with a prosthetic mesh can significantly reduce the post-operative recurrence rate. However, there are many mesh-related complications such as intestinal fistula caused by mesh erosion and intestinal obstruction resulting from adhesion of the mesh to the intestine (8–10). A laparoscopic approach (e.g., sugarbaker, keyhole, or sandwich procedures) combined with mesh repair has gained popularity in recent years for the treatment of parastomal hernia (10). However, this technique requires expensive anti-adhesion mesh with a specialized coating that still adheres to the intestines. Extraperitoneal mesh placement by laparoscopy can improve the surgical results and achieve a better clinical outcome while reducing the overall cost of treatment. The totally endoscopic sublay (TES) technique has been previously described (11) and extraperitoneal/sublay parastomal hernia repair has been explored in open, laparoscopic, endoscopic, and robotic surgeries (12–17). Here we present a case of a 74-year-old patient with parastomal hernia and a history of open radical resection treated with a totally extraperitoneal (TEP) endoscopic approach, with a good clinical outcome. We discuss the feasibility and possible operative approaches for endoscopic repair of parastomal hernia with the TEP technique.

## Case Report

A 74-year-old male patient visited our hospital in October 2020 with a mass above the stoma that had persisted for 2 months. He had no history of abdominal pain, fever, or cough. In 2016, he underwent an abdominoperineal resection procedure for carcinoma located in the distal one-third of the rectum. He also had a history of open appendectomy. The patient was an active smoker, was on medication for diabetes, had no chronic obstructive pulmonary disease, and was not using any immunosuppressive drugs. He had also received chemotherapy (10 cycles of FOLFOX6) for carcinoma. There was no history of any other comorbidities. Family, psychosocial, and genetic histories were insignificant.

### Physical Examination

The patient's blood pressure was 140/90 mmHg, heart rate was 82 beats/min, respiratory rate was 20 breaths/min, and body temperature was 36.4°C during the examination. His body mass index was 27.5 kg/m^2^. Abdominal examination in the erect position revealed a mass with a diameter of ~5 cm protruding above the stoma. He had midline and gridiron incisional scars. There was a parastomal hernia at the outer edge of the rectus abdominis muscle. The hernia was soft and reducible to the abdominal cavity in the supine position.

**Figure 1:**
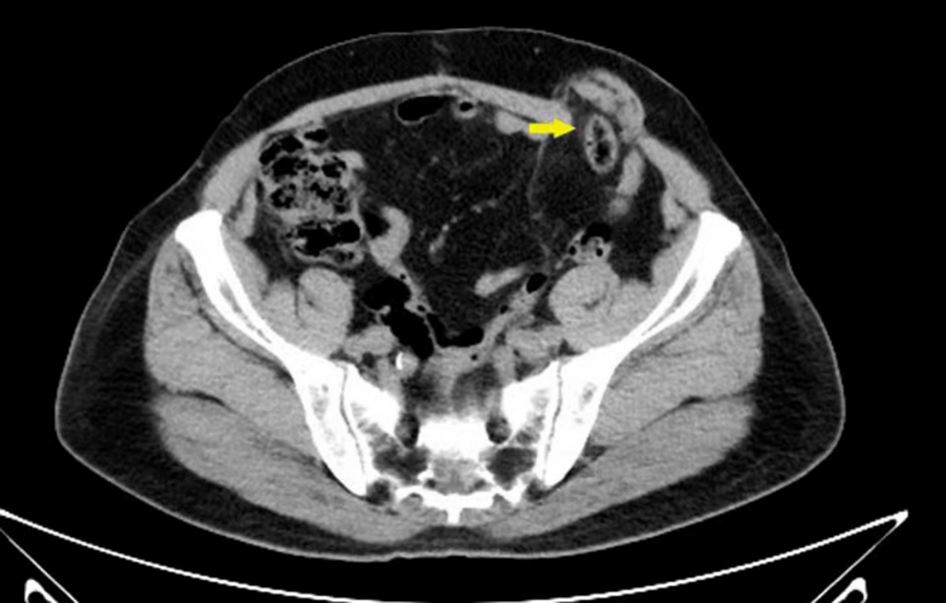
Pre-operative CT scan showing parastomal hernia (yellow arrow).

Computed tomography (CT) examination revealed a parastomal hernia (Figure 1) with a diameter of 3 cm. Laboratory tests revealed a leukocyte count of 5.90 × 10^9^/l, hemoglobin concentration of 65 g/l, neutrophil percentage of 61.8%, and platelet count of 149 × 10^9^/l. Glycosylated hemoglobin A1c (HbA1c) level was 6.5%. The renal function test showed that creatinine was 79.2 μmol/l and urea was 7.21 mmol/l. Electrolytes and coagulation function were normal. There were no abnormalities detected in the electrocardiogram, chest X-ray, and echocardiogram. The diagnosis at admission was post-operative rectal cancer with a parastomal hernia.

### Surgical Technique

Under general anesthesia, endotracheal intubation or a laryngeal mask and pre-operative indwelling urinary catheterization were applied to the patient, who was in a supine position. The surgeons were standing contralateral to the stoma with the monitor over the surgical site. The stoma was sealed with an adhesive incise drape. The surgical field was divided into right and left partitions using the sterilized surgical towel to prevent any potential contamination.

#### Step 1

**Figure 2:**
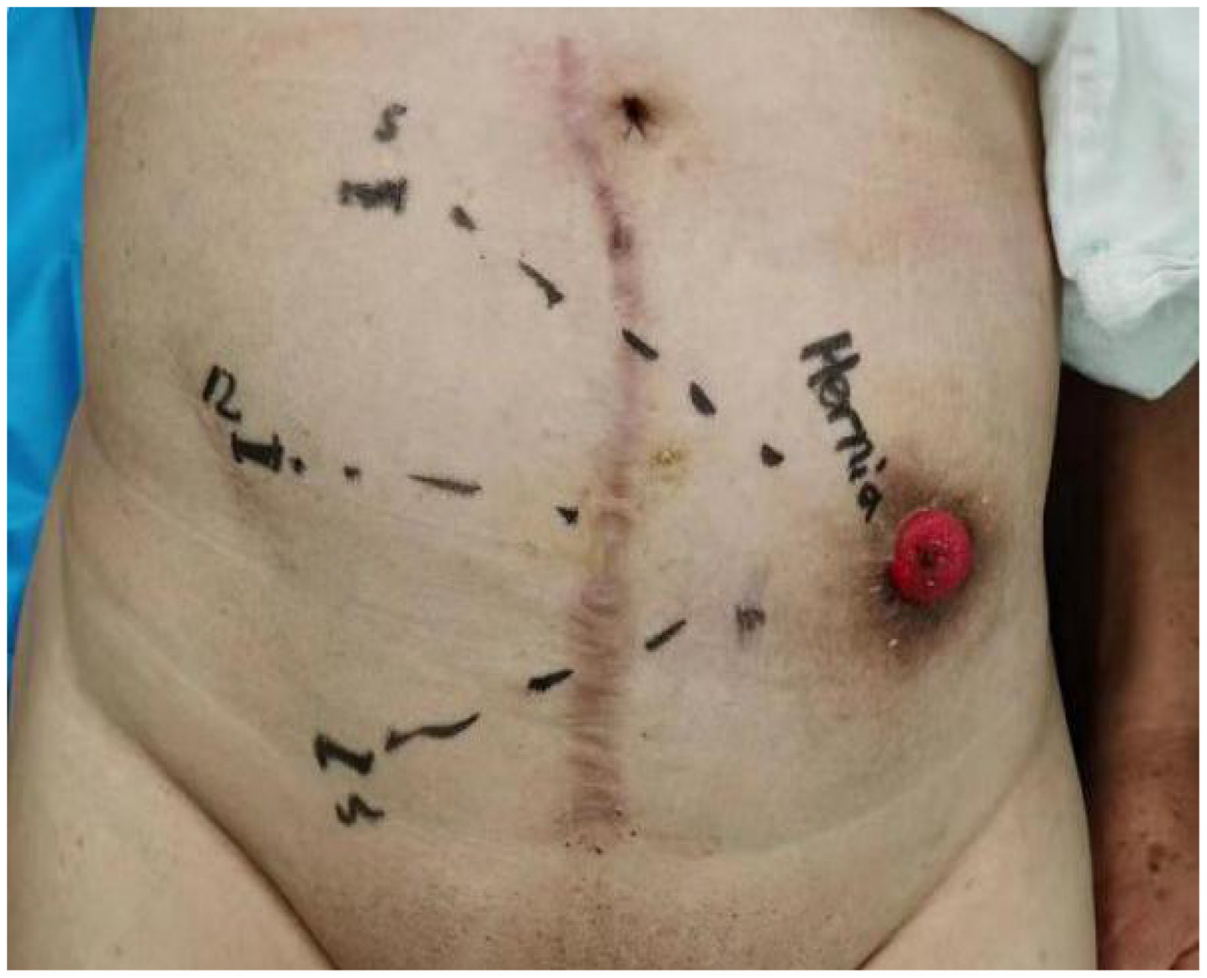
Layout of the trocar placement.

An incision of 1.2 cm through the skin and subcutaneous adipose tissue was made at the right iliac region. The incised subcutaneous tissue was separated using a pair of retractors to expose and dissect aponeurosis of the external oblique muscle. Hemostatic forceps were used to separate the internal oblique and transversus abdominis muscles. The primary extraperitoneal space was created using a finger. To create the preperitoneal space, 2 sets of 5-mm trocars were inserted at the site of surgical incision using the retrograde puncture technique (18); the trocars were placed at ~5 cm to each side of the incision, and a 12-mm observation trocar was placed through the incision (Figure 2). After successfully placing all three trocars, extrapneumoperitoneum was induced (11 mmHg CO_2_) for endoscope insertion.

#### Step 2

**Figure 3:**
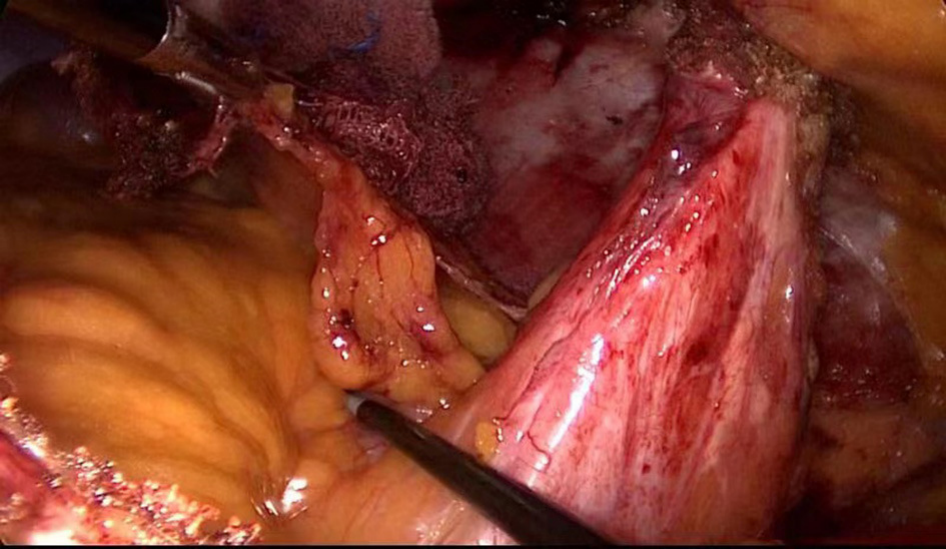
Anatomic relationship at the end of peritoneal dissociation.

The further extraperitoneal space was created under direct endoscopic guidance down to the pubic floor and up to the bladder, where it was easier to create. We then moved to the contralateral side of the abdomen; as the long midline incision in the abdomen made it very difficult to separate the peritoneum, the membrane was cut while avoiding damage to the tissue at the middle of the incision, thus weakening the abdominal wall. When performing cephalad dissection, we targeted the space between the rectus abdominis and posterior sheath. To create the space, we opened the lateral edge of the posterior sheath to penetrate the extraperitoneal space between the lateral and mid-abdomen. The TEP technique was used for this separation. The dissecting plane of the lateral space was the extraperitoneal space between the peritoneum and parietal plane. During separation, the peritoneum was dissected and observed to avoid damage to the bowel around the stoma. Through the space that was created by separation, we enlarged the extraperitoneal space within a 15- to 20-cm radius around the stoma, providing a large field for mesh placement (Figure 3).

#### Step 3

**Figure 4:**
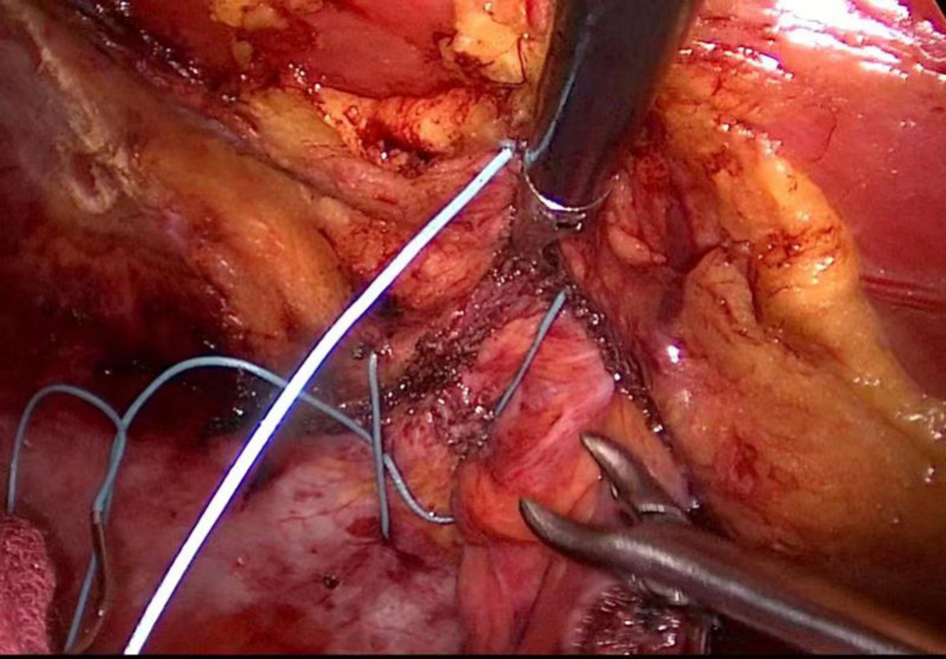
Intraoperative picture of hernia ring closure.

At the end of the procedure, the abdominal wall defect was closed with a 2-0 non-absorbable suture (Figure 4). The peritoneum defect was secured using a 3-0 absorbable suture with the enterostomy loop positioned outside the peritoneum.

#### Step 4

**Figure 5:**
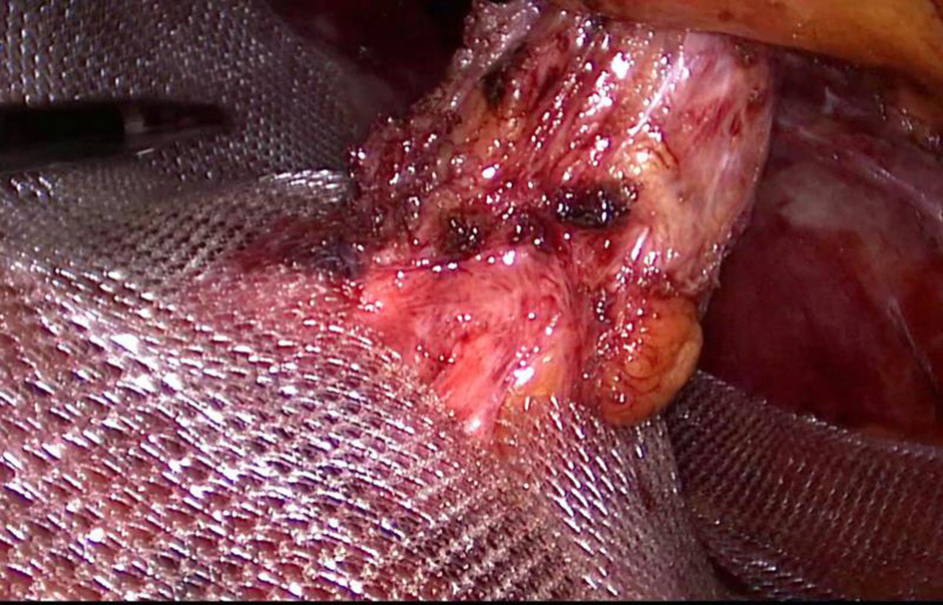
Mesh placement.

A 15 × 15-cm polypropylene mesh was cut in the middle and placed around the colostomy loop (Figure 5), then sutured and fixed. After a careful re-examination, the surgical field was clean, and no drainage tube was placed. Then extrapneumoperitoneum was relieved to complete the operation.

### Results

**Figure 6:**
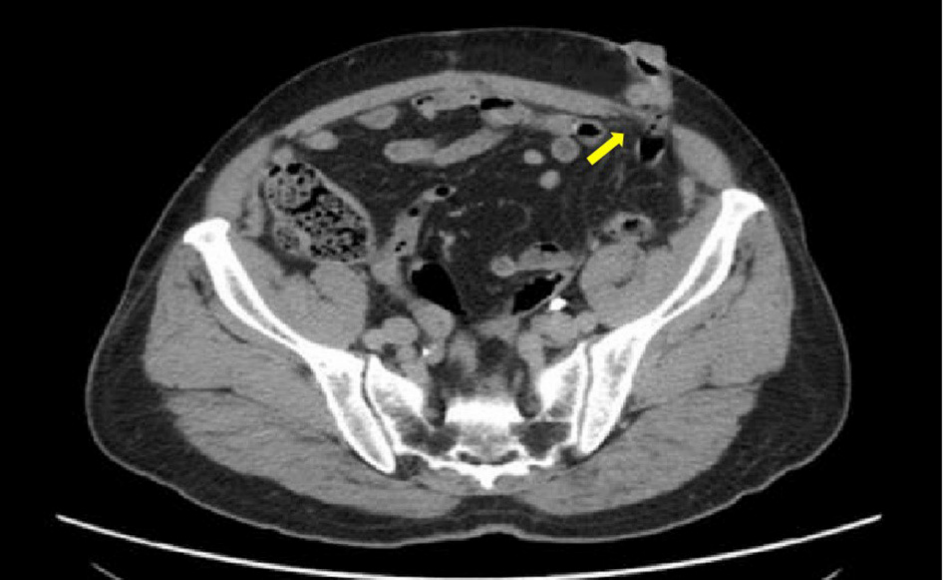
Post-operative CT image showing no recurrence of hernia (yellow arrow).

The total operation time was 240 min while the peritoneal separation time was 115 min. The patient was discharged 48 h after the operation. At the 3-month follow-up, the patient had a regular oral intake and defecation, no pain around the stoma, no seroma formation, and no infection at the incision site. The follow-up abdominal CT scan showed no recurrence of the hernia (Figure 6).

## Discussion

An intraperitoneal approach is typically used in laparoscopic parastomal hernia repair surgery, which involves the placement of an anti-adhesion mesh with a special coating that is in contact with the bowel. However, there is a risk of intestinal adhesion or fistula, and it can be life-threatening (19). TEP technique is already used in laparoscopic repair of many types of hernia, with good results (11, 20–22). We successfully separated the peritoneum by TEP endoscopy and repaired the parastomal hernia using synthetic mesh (without anti-adhesion coating). The difficulty of this operation was in the creation of extraperitoneal space around the stoma.

**Figure 7:**
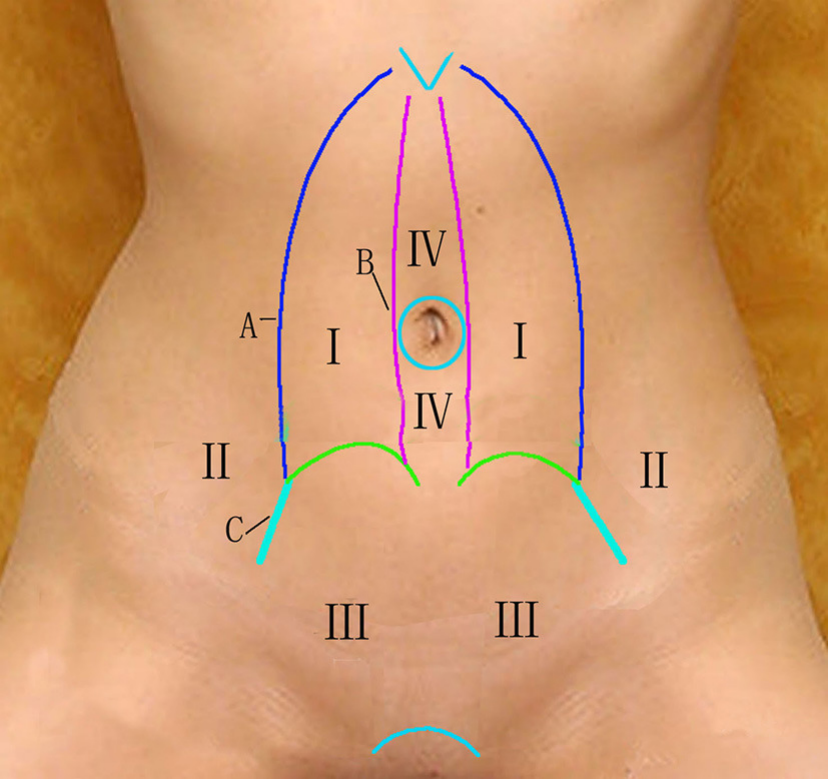
Abdominal wall area. (I) between the rectus abdominis and posterior sheath of rectus abdominis; (II) the Bogros space; (III) Retzius space, and (IV) between the linea alba and peritoneum. (A) between the lateral edge of the posterior sheath of the rectus abdominis and transverse abdominal muscle; (B) between the medial border of the posterior sheath of the rectus abdominis; and (C) between the transverse fascia of the abdomen extending downward and thickening at the outer edge of the arcuate line.

While operating, it is critical to move along the abdominal wall. On the abdominal wall, there are several easily separated spaces (Figure 7). Area I lies between the rectus abdominis and posterior sheath of rectus abdominis. Area II includes the Bogros space, which extends between the transversalis fascia and parietal peritoneum and is a commonly used zone in urologic surgery. Area III comprises the Retzius space (located posterior to transversalis fascia and anterior to the urinary bladder) that is an extension for TEP operation in the lower abdomen. Area IV, between the linea alba and peritoneum, is easy to separate because it contains a large amount of adipose tissue. This area is divided into upper and lower parts by the umbilical region, which also needs to be separated. There is no connection between these spaces because of their different anatomic levels. In order to connect all of the abdominal spaces, three major partitions and the umbilical area must be penetrated (11). The partitions are as follows: (A) between the lateral edge of the posterior sheath of the rectus abdominis and transverse abdominal muscle; (B) between the medial border of the posterior sheath of the rectus abdominis; and (C) between the transverse fascia of the abdomen extending downward and thickening at the outer edge of the arcuate line. This partitioning makes the separation of the extraperitoneal space very straightforward.

Two kind of separations could be performed in Area I—i.e., the rectus abdominis and posterior sheath, and the posterior sheath and peritoneum. The peritoneum is usually extremely thin and adheres tightly to and could not be separated from the posterior sheath. However, we attempted to confirm that the space between the peritoneum and posterior sheath can be separated, thus laying a foundation for TEP repair of parastomal hernia. The long midline incision on the abdomen in patients with a history of abdominal surgery makes it difficult to separate the peritoneum, which must be opened for the operation. Additionally, an open peritoneum allows observation of the intestine within the abdominal cavity, which can reduce the risk of accidental injury. Most hospitals have been able to implement laparoscopic transperineal rectal cancer surgery to reduce the use of abdominal incisions; this facilitates the creation of the extraperitoneal space and reduces the difficulty of the operation.

The mesh was placed after closing the defect at the end of the separation. We considered three ways to place the mesh, i.e., lap-TES-keyhole, lap-TES-sugarbaker, and lap-TES-sandwich (23–25). In our patient, the sigmoid colon was short, causing it to adhere to the ventral wall with a certain tension. We therefore selected the keyhole-like method. The sugarbaker method is considered optimal; when a sugarbaker-like placement is used, the bowel at the stoma is treated in the same manner as the spermatic cord in inguinal hernia surgery. As in the case of the lap-TES-keyhole method, it is possible that TEP sugarbaker and sandwich surgical methods will be established in the future.

In our case, the peritoneum separation time by laparoscopy was just 115 min, which was shorter than expected. With more patients and the accumulation of surgical experience, the operation time will decrease. The repair method of placing the mesh outside the peritoneum effectively avoids intestinal adhesion incidents and allows the use of ordinary mesh, thus reducing costs. At 48 h post-surgery, the patient was discharged with no post-operative complications such as pain, bleeding, or sepsis.

Repair of parastomal hernia was previously performed around the stoma. In that situation, the mesh was easily contaminated during placement by intestinal contents with high concentrations of local bacteria, thereby increasing the risk of infection (26). However, with the TEP endoscopic approach there is no surgical incision around the stoma, which reduces the risk of infection. Thus, the TEP method has several advantages over other surgical techniques for parastomal hernia repair.

Stoma necrosis, bowel obstruction, and/or perforation were observed during long-term follow-up in a study that used the retromuscular sugarbaker technique to repair a parastomal hernia (12). In our patient, there was no mesh-related complications up to 3 months post-surgery. The other study reported a high recurrence rate (37%) with the keyhole technique (27); however, a case–control prospective study found that surgical repair with the keyhole method using a polypropylene mesh with an anti-adhesive layer was superior to sugarbaker repair in terms of post-operative complications, recurrence rate, and mesh-related long-term morbidity (9). The lack of data on long-term outcomes and technical difficulties are some shortcomings of the TEP endoscopy technique; additionally, the clinical efficacy, recurrence rate, and late complications must be confirmed in more cases with long-term follow-up.

## Conclusion

Parastomal hernia repair using the TEP endoscopic approach is feasible, even in a patient who has previously undergone open rectal resection. Although long-term outcomes are unknown, this novel approach offers a new option for the surgical treatment of parastomal hernia.

## Data Availability Statement

The raw data supporting the conclusions of this article will be made available by the authors, without undue reservation.

## Ethics Statement

The studies involving human participants were reviewed and approved by Ethics committee of Northeast International Hospital. The patients/participants provided their written informed consent to participate in this study. Written informed consent was obtained from the individual(s) for the publication of any potentially identifiable images or data included in this article.

## Author Contributions

HJ and MW contributed to study conception and design and data acquisition and interpretation, and drafted the article. DT contributed to data analysis and interpretation, and drafted the article. CM and XC contributed to data acquisition and interpretation. All authors critically revised and approved the final version.

## Conflict of Interest

The authors declare that the research was conducted in the absence of any commercial or financial relationships that could be construed as a potential conflict of interest.
